# Post-COVID-19 Syndrome: Incidence, Risk Factor, and the Most Common Persisting Symptoms

**DOI:** 10.7759/cureus.32058

**Published:** 2022-11-30

**Authors:** Saad A Alghamdi, Mona A Alfares, Raeid A Alsulami, Abdullah F Alghamdi, Asim M Almalawi, Mohammed S Alghamdi, Hassan A Hazazi

**Affiliations:** 1 Infectious Diseases, King Abdulaziz University Hospital, Jeddah, SAU

**Keywords:** steroid use, fatigue, long-term health sequelae, post-covid syndrome, sars-cov-2, covid-19

## Abstract

Introduction: The pandemic of the coronavirus disease 2019 (COVID-19) has caused a significant burden worldwide. The most common presentation of coronavirus disease is acute, and most patients recover completely. However, now a substantial proportion of patients experience long-term health effects. Post-COVID-19 syndrome (PCS) is defined as “signs and symptoms that develop after an infection consistent with COVID-19 that persist for more than 12 weeks and have not been explained yet by an alternative diagnosis.” We faced a lack of studies regarding PCS in the Gulf area. Therefore, this study aimed to assess the incidence, risk factors, and most common persisting symptoms of PCS in confirmed COVID-19 patients who presented to King Abdulaziz University Hospital (KAUH) in Jeddah between June 1, 2020 and December 31, 2020.

Methods: This retrospective cohort study was conducted via telephone survey, which took place in June 2022 at KAUH. PCS was defined as the presence of one or more symptoms beyond 12 weeks from the onset of the illness. The inclusion criteria were patients aged 18 or above with laboratory-confirmed SARS-CoV-2 infection through positive RT-PCR in KAUH from June 1, 2020 to December 31, 2020, and both genders were included. The exclusion criteria were inability to provide informed consent, death, currently active COVID-19 infection (PCR +ve), and if they did not complete the interview. Medical records were obtained from patients diagnosed with COVID-19 through positive RT-PCR tests from June 1, 2020 to December 31, 2020.

Results: Data of 504 patients were analyzed. The incidence of PCS was 45.0% (95%CI, 40.7% to 49.5%). PCS was associated with female gender (OR = 1.71, 95%CI, 1.13 to 2.59, p = 0.011), having three or more co-morbid conditions (OR = 2.37, 95%CI, 1.19 to 4.75, p = 0.014), receiving steroids (OR = 2.13, 95%CI, 1.16 to 3.98, p = 0.016), also patients who experienced congestion (OR = 1.68, 95%CI, 1.05 to 2.71, p = 0.032) and depression (OR = 1.80, 95%CI, 1.03 to 3.18, p = 0.039) during acute COVID-19 infection. The most commonly reported symptoms beyond 12 weeks included fatigue (19.6%), joint pain (14.1%), and decreased exercise tolerance (12.7%).

Conclusion: In conclusion, the main risk factors to develop PCS are being female, having three or more co-morbidities, receiving steroids, or patients presenting with nasal congestion and/or depression.

## Introduction

The pandemic of the coronavirus disease 2019 (COVID-19) has caused a significant burden worldwide. The clinical spectrum of COVID-19 infections can range from asymptomatic infection to respiratory disease, multi-organ failure, and death [1]. One of the most serious issues with COVID-19 is its rapid spread; millions of people have been infected worldwide, and a rising number of deaths have been reported daily [2]. Patients experience various symptoms such as fever, dry cough, and fatigue, which are mild in about 80% of cases. However, the severity of the case may advance to develop respiratory distress or respiratory failure, necessitating the need to get admitted to the intensive care unit (ICU) [3,4]. COVID-19 has an acute presentation with patients recovering completely, but now a significant proportion of patients who recovered from acute infection have been experiencing long-term health effects [5-8]. The Patient-Led Research Collaborative, a citizen scientist group, first described post-COVID-19 syndrome (PCS) in a survey of prolonged COVID-19 symptoms conducted in the spring of 2020 [7]. The National Institute for Health and Care Excellence (NICE) defines long COVID-19, or PCS, as “signs and symptoms that manifest during or after an infection associated with COVID-19 that last beyond 12 weeks and are not justified by an alternate diagnosis” [9]. Symptoms can last for months and interfere with the patients' work and their quality of life [6,10].

Extensive research has shown that the incidence of PCS sequelae is estimated to be between 10% and 35% [11,12]. In a telephone survey of symptomatic patients with a positive outpatient test result for COVID-19 infection conducted in multiple states, 35% had not returned to their previous state of health when contacted two to three weeks following testing. In other words, one out of five adults aged 18-34 with no chronic health issues had not recovered to their normal state of health [12]. Another study found that only 10.8% of all subjects had no symptoms after recovering from the disease, while a large percentage of subjects had multiple symptoms [3]. The literature found that the common symptoms were anosmia, ageusia, fatigue, chest pain, or shortness of breath as PCS at months four and seven, respectively [8,13-15]. Another study found that the most commonly reported symptoms were fatigue (72.8%), anxiety (38%), joint pain (31.4%), continuous headache, and dementia [3]. As shown above, PCS appears to be a multi-system disease, occurring even after a relatively mild acute illness even among young adults without underlying chronic medical conditions [11,12].

To the best of our knowledge, no published studies were conducted regarding the incidence and precipitating factors of PCS in the Middle east. For this reason, the purpose of this research was to evaluate the incidence rate, risk factors, and the most common persistent symptoms of PCS in patients with confirmed COVID-19 infection tests at King Abdulaziz University Hospital (KAUH) in Jeddah, Saudi Arabia from June 2020 to December 2020.

## Materials and methods

**Aim and objective

This study aims to determine the incidence rate, risk factors, and most common persistent symptoms of PCS among hospitalized and non-hospitalized patients with confirmed SARS-CoV-2 infection tests conducted at KAUH in Jeddah, Saudi Arabia from June 1, 2020 to December 31, 2020.

**Study design and setting

This is a retrospective cohort study with a telephone survey that took place in June 2022. The medical records of patients with COVID-19 with laboratory-confirmed positive RT-PCR tests who were admitted to KAUH, a tertiary care center, were reviewed. Then follow-ups for these patients were conducted via telephone surveys to determine if they develop PCS symptoms. The study was conducted in the infectious disease department and approved by the Research Ethics Committee of KAUH. Reference No 7-22, (HA-02-J-008) number of Registration at National Committee of Bio. & Med. Ethics and informed oral consent were obtained from all participants.

Study population and sample size

The inclusion criteria included both genders, patients aged 18 or above, and patients with laboratory-confirmed SARS-CoV-2 infection through positive RT-PCR in KAUH from June 1, 2020 to December 31, 2020. The exclusion criteria were the inability to provide informed consent, death, currently active COVID-19 infection (PCR +ve), and if the patients did not complete the interview. Medical records of 6,749 who performed PCR tests were obtained from June 1, 2020 to December 31, 2020, out of which 1,408 records showed positive PCR tests for COVID-19. Five hundred fifty of them were unreachable, 153 died, 129 were less than 18 years old, 72 refused to participate and 504 of whom met the inclusion criteria were contacted to answer the survey via the telephone.

Data collection and definition of variables

COVID-19 was defined as a case with laboratory-confirmed SARS-CoV-2 infection as evidenced by positive RT-PCR in a patient with or without symptoms. The presence of one or more symptoms beyond 12 weeks from the onset of the illness was defined as a PCS. Files of patients admitted with COVID-19 from June 1, 2020 to December 31, 20200 were extracted from the hospital data system (Phoenix) by a trained medical student using google forms as a data collection method; it consists of demographic data including medical records number (MRN), age, gender, and nationality, and data on specific symptoms potentially correlated with COVID-19 including, systemic symptoms (fever, fatigue, joint pain), cardiorespiratory symptoms (cough, dyspnea, sore throat, congestion, chest pain, decreased exercise tolerance) gastrointestinal symptoms (abdominal pain, nausea, vomiting, diarrhea, constipation), CNS manifestations (loss of taste, loss of, smell, headache, hearing loss), psychiatric symptoms (depression, anxiety) complete all the variables. Moreover, pre-existing medical conditions such as HTN, DM, asthma, obesity, immunosuppressive condition, an autoimmune condition, psychiatric condition, a blood disorder, COPD, chronic kidney disease, liver disease, neurological condition, coronary artery disease, CHF, cancer, pregnancy, smoker were included. Also, ward or ICU admissions, length of hospital stay, and medication use such as steroids, antibiotics, tocilizumab, plasmapheresis, antiviral therapy, vitamin, and vaccination status were included. In addition to these, lab findings (blood workup, liver enzyme, urea, electrolyte, CPK, and inflammatory markers, e.g., CPR) were also noted. Patients were asked through telephone surveys in a close-ended question pattern, duration of each call took 10-15 minutes to recall the presence or absence of symptoms during the acute phase of COVID-19 and whether each symptom persisted. If symptoms persisted, they were then questioned about the duration of persistent symptoms, and the responses were documented.

Data entry and data analysis

Microsoft Excel 2020 was the program used for data entry. Statistical analysis was carried out using RStudio (v 4.1.1). Descriptive statistics were used to express categorical data (frequency and percentage) and continuous data (median and interquartile range [IQR]). Factors associated with the PCS were assessed using Pearson's Chi-squared test or Fisher's exact test for categorical variables and the Wilcoxon rank sum test for continuous variables. The significantly associated factors from the correlation analysis were subsequently entered into a multivariable logistic regression model. The outcomes of the regression analysis were expressed as odds ratio (OR) and the respective 95% confidence intervals (95%CI). Statistical significance was considered at p < 0.05.

## Results

Demographic characteristics and the history of co-morbidities

Data of 504 patients were analyzed. More than half of the patients were males (52.6%) and Saudis (55.8%), and less than half of them (48.2%) were aged >30 to 50 years. The most reported co-morbidities included diabetes (26.8%), hypertension (25.6%), and obesity (23.0%). More than one-quarter of patients (28.9%) had one comorbidity, whereas 17.1% and 19.9% of them had two or ≥3 co-morbidities, respectively (Table 1).

**Table 1:** Demographic characteristics and the history of comorbidities and their association with the incidence of post-COVID-19 syndrome.

Parameter	Category	Overall, N = 504	Post-COVID-19 syndrome		
			No, N = 277	Yes, N = 227	p
Gender	Male	265 (52.6%)	167 (63.0%)	98 (37.0%)	<0.0001
	Female	239 (47.4%)	110 (46.0%)	129 (54.0%)	
Age	18-30 y	78 (15.5%)	46 (16.6%)	32 (14.1%)	0.549
	>30-50 y	243 (48.2%)	138 (49.8%)	105 (46.3%)	
	>50-70 y	153 (30.4%)	78 (28.2%)	75 (33.0%)	
	>70 y	30 (6.0%)	15 (5.4%)	15 (6.6%)	
Nationality	Saudi	281 (55.8%)	145 (51.6%)	136 (48.4%)	0.089
	Non-Saudi	223 (44.2%)	132 (59.2%)	91 (40.8%)	
Comorbidities	Asthma	38 (7.6%)	14 (5.1%)	24 (10.6%)	0.020
	HTN	129 (25.6%)	63 (22.7%)	66 (29.2%)	0.099
	DM	135 (26.8%)	66 (23.8%)	69 (30.5%)	0.091
	Obesity	114 (23.0%)	56 (20.6%)	58 (26.0%)	0.154
	Smoking (current or former)	103 (20.5%)	48 (17.3%)	55 (24.3%)	0.053
	COPD	8 (1.6%)	1 (0.4%)	7 (3.1%)	0.025
	CAD	21 (4.2%)	8 (2.9%)	13 (5.8%)	0.110
	CHF	11 (2.2%)	4 (1.5%)	7 (3.1%)	0.236
	Pregnancy	11 (4.7%)	4 (3.7%)	7 (5.6%)	0.550
	Liver disease	4 (0.8%)	1 (0.4%)	3 (1.3%)	0.331
	CKD	17 (3.4%)	9 (3.3%)	8 (3.5%)	0.864
	Autoimmune condition	6 (1.2%)	2 (0.7%)	4 (1.8%)	0.416
	Immunosuppression	6 (1.2%)	2 (0.7%)	4 (1.8%)	0.416
	Blood disorder	27 (5.4%)	10 (3.6%)	17 (7.5%)	0.054
	Neurological condition	13 (2.6%)	7 (2.5%)	6 (2.7%)	0.934
	Dyslipidemia	90 (17.9%)	42 (15.2%)	48 (21.3%)	0.073
No. of Medical comorbidities	None	171 (34.1%)	112 (40.6%)	59 (26.1%)	0.002
	1	145 (28.9%)	78 (28.3%)	67 (29.6%)	
	2	86 (17.1%)	43 (15.6%)	43 (19.0%)	
	>=3	100 (19.9%)	43 (15.6%)	57 (25.2%)	

Clinical characteristics

In general, only 6.5% of patients had received the COVID-19 vaccine, and 14.1% had received the seasonal influenza vaccine before acquiring the initial infection. The most used medications were vitamin supplements (59.7%), antibiotics (41.1%), and other drugs such as medications for diabetes mellitus and hypertension (32.7%). Upon the initial diagnosis of COVID-19, the most frequently reported symptoms were fever (80.5%), fatigue (79.9%), and joint pain (64.4%). A total of 177 patients (35.2%) were admitted to a medical ward, and 40 patients (8.0%) were admitted to an ICU (Table 2). Regarding the laboratory parameters, descriptive data are presented in Table 3.

**Table 2:** Clinical characteristics of patients and their association with the incidence of post-COVID-19 syndrome. *Data are presented as median (IQR) based on a subgroup of patients (n=217) who were admitted to either a medical ward or ICU. Otherwise, data are expressed as frequency (percentage).

Parameter	Category	Overall, N = 504	Post-COVID-19 syndrome		
			No, N = 277	Yes, N = 227	p
History of medications	Vitamin supplements	301 (59.7%)	156 (56.3%)	145 (63.9%)	0.085
	Steroid	102 (20.2%)	39 (14.1%)	63 (27.8%)	<0.001
	Antibiotic	207 (41.1%)	107 (38.6%)	100 (44.1%)	0.218
	Tocilizumab	15 (3.0%)	7 (2.5%)	8 (3.5%)	0.512
	Plasmapheresis	3 (0.6%)	1 (0.4%)	2 (0.9%)	0.591
	Anti-Viral Therapy	135 (26.8%)	73 (26.4%)	62 (27.3%)	0.809
	Others	165 (32.7%)	74 (26.7%)	91 (40.1%)	0.001
History of vaccination before infection	COVID-19 vaccine	33 (6.5%)	17 (6.1%)	16 (7.0%)	0.681
	Influenza vaccine	68 (14.1%)	33 (12.5%)	35 (16.1%)	0.256
COVID-19 symptoms on initial diagnosis	Headache	321 (63.8%)	156 (56.3%)	165 (73.0%)	<0.001
	Fever	405 (80.5%)	216 (78.0%)	189 (83.6%)	0.112
	Fatigue	402 (79.9%)	204 (73.6%)	198 (87.6%)	<0.001
	Joint pain	324 (64.4%)	159 (57.4%)	165 (73.0%)	<0.001
	Cough	296 (58.8%)	145 (52.3%)	151 (66.8%)	0.001
	Congestion	190 (37.8%)	83 (30.0%)	107 (47.3%)	<0.001
	Sore throat	236 (46.9%)	116 (41.9%)	120 (53.1%)	0.012
	Dyspnea	227 (45.1%)	96 (34.7%)	131 (58.0%)	<0.001
	Chest pain	139 (27.6%)	52 (18.8%)	87 (38.5%)	<0.001
	Loss of taste	304 (60.4%)	148 (53.4%)	156 (69.0%)	<0.001
	Loss of smell	316 (62.8%)	151 (54.5%)	165 (73.0%)	<0.001
	Hearing loss	23 (4.6%)	7 (2.5%)	16 (7.1%)	0.015
	Nausea	131 (26.0%)	53 (19.1%)	78 (34.5%)	<0.001
	Vomiting	89 (17.7%)	36 (13.0%)	53 (23.5%)	0.002
	Abdominal pain	78 (15.5%)	31 (11.2%)	47 (20.8%)	0.003
	Diarrhea	145 (28.8%)	68 (24.5%)	77 (34.1%)	0.019
	Constipation	29 (5.8%)	9 (3.2%)	20 (8.8%)	0.007
	Decreased exercise tolerance	309 (61.4%)	150 (54.2%)	159 (70.4%)	<0.001
	Depression	131 (26.0%)	48 (17.3%)	83 (36.7%)	<0.001
	Anxiety	154 (30.6%)	65 (23.5%)	89 (39.4%)	<0.001
Hospital admission	No admission	286 (56.9%)	174 (62.8%)	112 (49.6%)	0.001
	Medical ward	177 (35.2%)	90 (32.5%)	87 (38.5%)	
	ICU	40 (8.0%)	13 (4.7%)	27 (11.9%)	
Length of hospital stay*	Days	7.0 (3.0, 14.0)	7.0 (3.0, 14.0)	8.0 (4.2, 14.0)	0.603

**Table 3:** Descriptive statistics of the laboratory parameters among the patients. *The frequency of missing records is presented as follows: N of missing records in the overall cohort (N of missing records among patients without post-COVID-19 syndrome/N of missing records among patients with post-COVID-19 syndrome). ¥ Data are expressed as median (interquartile range).

Parameter	Missing, N*	Overall, N = 504^¥^	Post-COVID-19 syndrome		
			No, N = 277^¥^	Yes, N = 227^¥^	p
WBCs	269 (164/105)	5.8 (4.5, 7.8)	6.0 (4.5, 7.9)	5.6 (4.6, 7.7)	0.833
Hgb	267 (162/105)	13.0 (11.6, 14.2)	12.9 (11.1, 14.1)	13.0 (12.0, 14.4)	0.114
Platelet count	267 (163/104)	225.0 (183.0, 300.0)	226.0 (183.8, 300.0)	224.0 (183.0, 295.5)	0.626
Urea	275 (165/110)	4.0 (3.1, 5.6)	4.2 (3.1, 5.7)	4.0 (3.1, 5.3)	0.884
Creatinine	271 (164/107)	73.0 (60.0, 90.0)	75.0 (61.0, 92.0)	72.0 (60.0, 86.3)	0.344
AST	321 (190/131)	29.0 (19.0, 49.5)	29.0 (20.0, 53.0)	30.0 (18.8, 46.2)	0.669
ALT	294 (174/120)	34.0 (23.0, 54.0)	34.0 (25.0, 52.5)	33.0 (20.5, 55.0)	0.683
CRP	317 (186/131)	31.0 (9.9, 102.5)	38.5 (12.2, 104.0)	22.1 (6.4, 97.9)	0.116
CPK	368 (210/158)	101.5 (53.8, 188.8)	98.0 (58.5, 270.5)	104.0 (51.0, 165.0)	0.283

Characteristics of PCS

**Figure 1:**
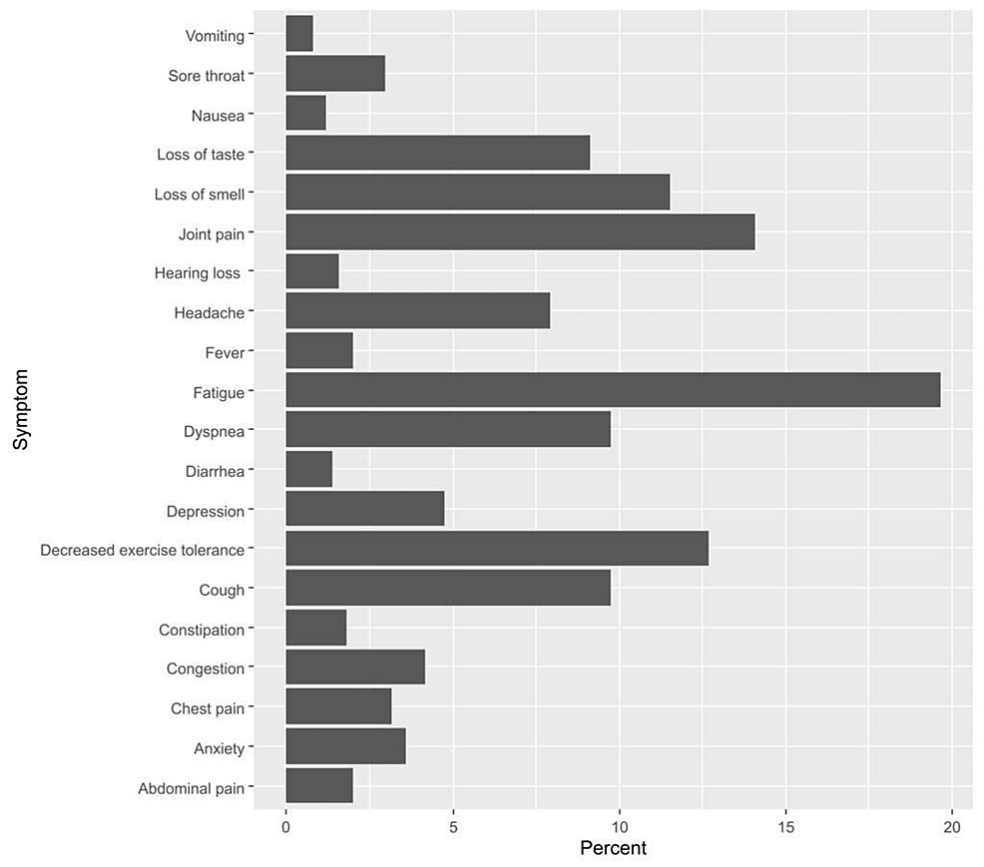
Persistent symptoms of post-COVID-19 syndrome

Out of the 504 patients, 227 had reported at least one symptom that developed during or after infection and persisted beyond 12 weeks; therefore, the incidence of PCS was 45.0% (95%CI, 40.7% to 49.5%). The most reported symptoms beyond 12 weeks included fatigue (19.6%), joint pain (14.1%), and decreased exercise tolerance (12.7%) (Figure 1).

Factors associated with PCS

Compared to their counterparts, the incidence of PCS was significantly higher among females (54.0% vs 46.0% among males, p < 0.0001), as well as patients with a history of asthma (10.6% vs 5.1%, p = 0.020) and COPD (3.1% vs 0.4%, p = 0.020). Additionally, the incidence of long COVID-19 symptoms differed significantly according to the number of co-morbidities (p = 0.002) (Table 1). The incidence of PCS was also associated with receiving steroids (27.8% vs. 14.1%, p < 0.0001) and other medications (40.1% vs 26.7%, p = 0.001). Having persistent COVID-19 symptoms was significantly associated with ICU admission (11.9% vs 4.7% who had no PCS, p = 0.001). All the initial COVID-19 symptoms under investigation were significantly associated with the development and persistence of long COVID syndrome (except fever). More details about these symptoms are demonstrated in (Table 2). Concerning the laboratory parameters, none of the collected parameters were associated with PCS (Table 3).

Independent risk factors for the PCS

Results of the regression analysis revealed that the PCS was independently associated with the female gender (OR = 1.71, 95%CI, 1.13 to 2.59, p = 0.011), having three co-morbid conditions or more (OR = 2.37, 95%CI, 1.19 to 4.75, p = 0.014), and receiving steroids (OR = 2.13, 95%CI, 1.16 to 3.98, p = 0.016), as well as experiencing the following symptoms during the initial COVID-19 infection: congestion (OR = 1.68, 95%CI, 1.05 to 2.71, p = 0.032) and depression (OR = 1.80, 95%CI, 1.03 to 3.18, p = 0.039) (Table 4).

**Table 4:** Risk factors for post-COVID-19 syndrome.

Parameter	Category	OR	95% CI	P-value
Gender	Male	—	—	—
	Female	1.71	1.13, 2.59	0.011
Asthma	No	—	—	—
	Yes	1.20	0.53, 2.77	0.656
COPD	No	—	—	—
	Yes	4.74	0.59, 104	0.199
No. of Medical Condition	None	—	—	—
	1	1.66	0.98, 2.83	0.061
	2	1.72	0.89, 3.33	0.108
	>=3	2.37	1.19, 4.75	0.014
Steroid	No	—	—	—
	Yes	2.13	1.16, 3.98	0.016
Other medications	No	—	—	—
	Yes	0.96	0.55, 1.65	0.881
Hospital admission	No admission	—	—	—
	Medical ward	0.75	0.44, 1.27	0.285
	ICU	1.30	0.52, 3.30	0.570
Headache	No	—	—	—
	Yes	1.56	0.99, 2.46	0.052
Fatigue	No	—	—	—
	Yes	1.35	0.74, 2.48	0.326
Joint pain	No	—	—	—
	Yes	1.13	0.70, 1.82	0.613
Cough	No	—	—	—
	Yes	1.20	0.75, 1.92	0.442
Congestion	No	—	—	—
	Yes	1.68	1.05, 2.71	0.032
Sore throat	No	—	—	—
	Yes	0.79	0.49, 1.28	0.342
Dyspnea	No	—	—	—
	Yes	1.19	0.73, 1.94	0.486
Chest pain	No	—	—	—
	Yes	1.41	0.83, 2.37	0.200
Loss of taste	No	—	—	—
	Yes	0.95	0.47, 1.90	0.891
Loss of smell	No	—	—	—
	Yes	1.70	0.85, 3.44	0.135
Hearing loss	No	—	—	—
	Yes	1.63	0.60, 4.77	0.348
Nausea	No	—	—	—
	Yes	1.18	0.63, 2.21	0.600
Vomiting	No	—	—	—
	Yes	1.00	0.49, 2.04	>0.999
Abdominal pain	No	—	—	—
	Yes	1.17	0.63, 2.17	0.625
Diarrhea	No	—	—	—
	Yes	1.04	0.64, 1.69	0.873
Constipation	No	—	—	—
	Yes	1.09	0.44, 2.84	0.857
Decreased exercise tolerance	No	—	—	—
	Yes	0.84	0.52, 1.33	0.452
Depression	No	—	—	—
	Yes	1.80	1.03, 3.18	0.039
Anxiety	No	—	—	—
	Yes	1.20	0.71, 2.03	0.487

## Discussion

The present study set out to explore the incidence rate, risk factors, and the most common persisting symptoms of PCS in patients with laboratory-confirmed COVID-19 infection through a positive RT-PCR test at KAUH In Jeddah, Saudi Arabia.

Demographics and incidence of PCS

The majority of our participants were males, Saudi Arabians, and aged between 30 to 50 years. Moreover, a study done in Egypt by Kamal et al. demonstrated that half of the sample size was aged 31- 40, and the majority were females [3]. On the contrary, a study conducted in Turkey by Kayaaslan et al. found that the majority of participants were males over the age of 50 [16]. The younger age in this study may be due to the demographic distribution in Saudi Arabia [17]. The current investigation verified that patients with COVID-19 had a significant incidence of PCS (about 45%). This finding seems to be consistent with a previous prospective study conducted in Bangladesh which revealed that the incidence of PCS was 46% [18]. Moreover, the incidence was 50.9% in the prospective study of Moreno-Pérez et al. [19]. On the other hand, according to another review conducted in France, approximately 65% of COVID-19 patients stated that at least one symptom persisted even after two months after the initial infection [20]. We believe that the reasons behind the incidence variation are multifactorial, including the aspect of the research population (e.g., hospitalized vs outpatients, variations in the progression of the patient's illness), data collection method (e.g., electronic questionnaire, review of readmission and outpatient visit data), or the period since an acute infection [21].

Common reported symptoms with PCS

Distinct studies have documented a variety of symptoms of PCS. This study supports evidence from previous investigations which found that fatigue was by far, the most commonly reported symptom [3,8,22]. Furthermore, our analysis showed that joint pain (14.1%) and decreased exercise tolerance (12.7%) came in second place, whereas dyspnea and neurological symptoms were predominant in other findings [8,22]. The explanation for why fatigue predominated is unknown. This result may be explained as an alteration in the immune system due to viral infection [19,23]. Joint pain and decreased exercise tolerance can be interpreted by prolonged immobilization during the attack and recovery phase, hence almost half of our COVID-19 patients are hospitalized.

Factors associated with PCS

Except for the fever, all the initial COVID-19 symptoms were significantly associated with the development and persistence of PCS. These results agree with those of Augustin et al. and Mahmud et al. [5,18]. Additionally, the incidence of PCS symptoms increased significantly according to the number of co-morbidities (p = 0.002), such as a history of asthma and COPD. These results match those observed in earlier studies by Kamal et al. and Pavli et al. [3,8]. This result may be justified by the fact that any increase in co-morbidities, particularly chronic respiratory diseases, impair patients’ ability to fully recover. An unexpected finding was that receiving chronic disease medications (p = 0.001) was associated with the incidence of PCS. In reviewing the literature, we found no data to support the association between chronic disease medications and PCS [3,8,23]. Therefore, further work is required to establish the viability of these results and assess each drug independently. 

Independent risk factors for PCS

Our findings show that the female gender is more likely to be directly affected by PCS, which is consistent with previously published data from Barsky et al., Augustin et al., and Mahmud et al. It may be possible because females are more inclined to disclose their symptoms to a doctor [5,18,24]. Surprisingly, receiving steroids was identified to be an independent risk factor for PCS (p < 0.0001); these findings, however, have not previously been described. It is, therefore, likely that such connections exist because corticosteroids have potential immunosuppressive consequences that may facilitate viral replication and delayed viral clearance [25,26]. According to earlier findings of a prospective cohort study carried out in Bangladesh, they noticed that respiratory distress, lethargy, long duration of illness, and moderate severity of the disease is the major risk factor for PCS [18]. In addition, another prospective cohort research, by Augustin et al., demonstrated that anosmia and diarrhea during acute COVID-19 were independent predictors for PCS with an AOR of 5.12 and 2.35, respectively [5]. Considering the above, our analysis concluded that experiencing congestion and depression during the initial COVID-19 infection as well as, having three or more co-morbid conditions, were independent risk factors for PCS. These seemingly contradictory findings could be attributed to the influence of multiple factors such as residual inflammation during the convalescent phase, organ damage, non-specific effects from prolonged ventilation, such as post-intensive care syndrome, prolonged hospitalization, and social isolation, or impacts on underlying medical conditions [27,28]. 

Clinical characteristics of COVID-19

After evaluating COVID-19 symptoms during the infection, we found that the most frequently reported symptoms were fever, fatigue, and joint pain. Several reports have shown that the most commonly reported symptoms differ from the ones we had, for example, a longitudinal prospective cohort study by Augustin et al. showed that cough was the highest among the symptoms followed by ageusia, then anosmia [5]. Where bidirectional cohort research by Peghin et al. Stated that the most common symptom was fever, anosmia/dysgeusia, and cough respectively [23]. Due to the high occurrence of fever in COVID-19 patients, we recommend that fever should be managed closely in COVID-19 patients to reduce the burden of the disease. As for the aspect of co-morbid diseases, we stated that more than half of patients who were suffering from COVID-19 infection had at least one co-morbidity, another study done in Bangladesh by Mahmud et al. [18] revealed that one-third of patients have reported co-morbid diseases during their COVID-19 infection. Also, in the study of Kayaaslan et al. [16]. Less than half of the patients had at least one co-morbid disease during their infection period. Hence, the increased number of reported co-morbidities in our study may be explained by the fact that we considered obesity and smoking as co-morbid factors. Additionally, most of the co-morbidities we found were diabetes mellitus (26.8%), hypertension (25.6%), and obesity (23.0%) respectively. However, another study by Kayaaslan et al. showed the most common co-morbidities were hypertension, diabetes, and coronary artery disease of which corresponding percentages (20.3%), (15.4%), and (7.0%), respectively [16]. And a study done by Augustin et al. in Germany, showed hypertension of (8.0%), chronic lung disease (2.7%), and malignancies (2.4%) were the most reported co-morbidities [5]. And a study by Kamal et al. showed that (7.7%) have hypertension and (5.2%) have diabetes mellitus [3]. This high percentage of diabetes mellitus in our study may reflect its high prevalence in the Saudi population [17]. 

Hospital stays

The admission rate of COVID-19 infection recorded one-third of cases to the medical ward and less than 10% of cases to the ICU. The rest of the patients were home quarantined or managed as outpatient cases. Compared with Peghin et al. admission rates to the medical ward and ICU admission nearly matched the admission rates in our study [23]. On the other hand, Kayaaslan et al. study mentioned that the total admission rate of COVID-19 patients to the medical ward and the ICU follow-up were slightly higher than our study [16]. Hence, most of the patients did not require hospital admission; this might indicate that the virus’s danger does not lie in its virulence but rather in its infectivity.

Laboratory parameters**

Regarding the aspect of laboratory analysis, we found that there is no relation between lab parameters and PCS. These findings are consistent with Moreno-Pérez et al., who found no significant association in the laboratory parameter; the mutual parameter was the C-reactive protein [19]. As a result, laboratory parameters have almost no prognostic value for the PCS.

COVID-19 and influenza vaccine

In general, only 33 patients out of a total sample size of 504 received the COVID-19 vaccination, while 71 received the seasonal influenza vaccine before they got infected. Regarding the low percentage of COVID-19 vaccines taken by the patients, the reason behind that was the limited availability of the vaccine during that period. Judging the flu vaccine; we require more research discussing it among PCS patients. 

Limitation 

Our study had limitations since we were unable to include all laboratory markers such as IgG, SARS-CvV-2 Titer, and D-dimers, they were not routinely measured. Another limitation is the study was conducted in a single health facility, moreover, lack of published data from countrywide COVID-19 registries with long-term follow-up. Besides that, recall bias should be considered. Therefore, continuous documentation and standardized follow-up of COVID-19 patients are, thus, required to assess the clinical outcome in patients with PCS.

## Conclusions

The incidence of PCS was 45% in patients who recovered from COVID-19 infection. The most obvious risk factors to emerge from this study to develop a PCS are being a female, having three co-morbidities or more, receiving steroids, or patients presenting with congestion and/or depression. The most commonly reported symptoms are fatigue, joint pain, and decreased exercise tolerance. 

As the result shows, we recommend a further study to assess the long-term burden of post-COVID-19. Patients should receive medical attention from their medical physician in an outpatient clinic, considering any current or developing co-morbidities to help ensure that the appropriate healthcare services are offered. This finding has provided a deeper insight into establishing a guideline for the diagnosis and treatment of PCS based on specified standards necessary.
